# UnReal? Investigating the Sense of Reality and Psychotic Symptoms with Virtual Reality

**DOI:** 10.3390/jcm9061627

**Published:** 2020-05-28

**Authors:** Gad Drori, Paz Bar-Tal, Yonatan Stern, Yair Zvilichovsky, Roy Salomon

**Affiliations:** 1 Gonda Brain Research Center, Bar-Ilan University, Ramat Gan 5290002, Israel; bartalpaz@gmail.com (P.B.-T.); yoniastern@gmail.com (Y.S.); zvilich@gmail.com (Y.Z.); roy.salomon@biu.ac.il (R.S.); 2 Psychology Department, University of Haifa, Haifa 3498838, Israel

**Keywords:** sense of reality, virtual reality, hallucinations, psychosis, derealization

## Abstract

Distortions of reality, such as hallucinations, are common symptoms of many psychiatric conditions. Accordingly, sense of reality (SoR), the ability to discriminate between true and false perceptions, is a central criterion in the assessment of neurological and psychiatric health. Despite the critical role of the SoR in daily life, little is known about how this is formed in the mind. Here, we propose a novel theoretical and methodological framework to study the SoR and its relation to psychotic symptoms. In two experiments, we employed a specialized immersive virtual reality (VR) environment allowing for well-controlled manipulations of visual reality. We first tested the impact of manipulating visual reality on objective perceptual thresholds (just noticeable differences). In a second experiment, we tested how these manipulations affected subjective judgments of reality. The results revealed that the objective perceptual thresholds were robust and replicable, demonstrating that SoR is a stable psychometric property that can be measured experimentally. Furthermore, reality alterations reduced subjective reality judgments across all manipulated visual aspects. Finally, reduced sensitivity to changes in visual reality was related to self-reported prodromal psychotic symptoms. These results provide evidence for the relevance of SoR in the assessment of psychosis and other mental disorders in which reality is distorted.

## 1. Introduction

### 1.1. Sense of Reality

We normally and intuitively trust our sensory representation of the world to closely correspond to what “is really there” [1,2]. We term this correspondence “reality”, and differentiate it from other states in which our representations of the world do not match the environment, such as dreaming and hallucinations. Intriguingly, we seem to possess a capacity to judge whether our sensory experience corresponds to the world or not, i.e., a sense of reality (SoR). This capacity is a critical aspect of the human mind, allowing us to guide our actions based on meaningful sensory signals. Abnormal SoR processing may lead to a disparity between experience and reality, resulting in hallucinations (false perceptions), which is a core symptom of psychosis. However, while SoR is an important criterion in the assessment of neurological and psychiatric health [3,4,5], we know little regarding its underlying cognitive mechanisms. 

Previous work has focused on the mechanisms allowing the separation of internally and externally generated information in memory (i.e., source monitoring) (e.g., [6,7]). Source monitoring paradigms typically evaluate the ability to remember the source of a stimuli (e.g., was this word previously seen or imagined?) (e.g., [8]). This research has been based on the proposition that hallucinations are grounded in failures to discriminate the source of the information correctly (i.e., perception vs. imagery or memory). Thus, while the discrimination of imagery, perception, and memory has been studied extensively (e.g., [5,9,10]), the ability to discriminate between real and false perceptions in real time (i.e., perceptual reality monitoring) is poorly understood. This discrimination is essential as distortions of perceptual reality in the form of hallucinations or illusions originating from neurological, psychiatric, pharmacological, medical, or psychological origins are commonplace [11,12]. Despite the importance of this issue, there is scarce knowledge regarding ‘how do we decide what is real?’, or in other words, ‘how do we form a SoR?’

### 1.2. A Theoretical Framework of SoR

There is growing evidence that perception is an inferential process [13,14,15]. For instance, “predictive coding” frameworks suggest that perception arises through a process of inferences (*predictions*) about the likely causes of sensory information (i.e., *likelihood*). These *predictions* are based on previous experience, through which higher levels in the hierarchy attempt to predict the signals arising from the lower levels [14,16,17]. When an unusual sensory event (e.g., a pink elephant) violates predictions, a *prediction error* arises and propagates through the hierarchy until it is either “predicted away” at higher levels (e.g., I’m wearing pink glasses) or the generative model is updated (e.g., pink elephants exist) [15,18]. Perception is thus a process of inference based on our experientially acquired model of the world. Therefore, we suggest that SoR can be viewed as a probabilistic inference based on the magnitude of prediction errors expressing the fit between a given sensory signal and our model of the world [19,20]. For a given sensory event, the magnitude of the prediction error is the probability of the perception being “real” in light of one’s model of the world. Thus, viewing a “pink elephant” could be taken as a veridical perception, an optical aberration, or a hallucination, based on one’s model of the world.

### 1.3. Failures of SoR as a Conceptual Framework

Hallucinations (i.e., false perceptions) are a perplexing symptom of many psychiatric and neurological disorders. Advances in neuroscience and computational psychiatry relate hallucinations to breakdowns of predictive processes [20,21,22]. For example, overreliance on predictions, favoring prior expectations over sensory evidence, may lead to hallucinations [21,23,24]. On the other hand, the overweighting of sensory signals and deficient predictive processes, enabling the structuring of experience, may cause a sense of alienation from one’s own actions and thoughts, causing passivity symptoms [25,26,27]. 

Intriguingly, hallucinations can be experienced with or without insight into their nature as false perceptions. For example, patients with Charles Bonnet syndrome experience complex hallucinations yet typically identify these as hallucinations [28]. Contrarily, psychotic patients often lose the ability to discern between real and hallucinatory percepts, which has been linked to poorer prognosis [29] and reduced cognitive abilities [30,31]. Thus, psychiatric hallucinations and pseudo-hallucinations include a sensory aspect (i.e., unusual or non-veridical perceptual experience) and a metacognitive aspect related to insight (i.e., assessment of the validity of the perceptual experience). Indeed, depersonalization and derealization syndromes found in several psychiatric conditions produce a sense of “unrealness” in the absence of hallucinations, suggesting a deficit in SoR without the aberrant perceptual experience [32,33]. Thus, failures of the sense of reality leading to hallucinations, depersonalization, and derealization may be driven by either abnormal prediction error signaling impacting bottom-up sensory processing, or top-down predictions affecting the subjective experience of “unrealness“, or a combination of these two. Critically, psychosis typically presents both abnormal sensory experiences as well as diminished insight regarding the implausibility of these experiences. These various manifestations of psychopathology highlight the need for a better understanding of the different components of SoR.

### 1.4. Modeling SoR Using Virtual Reality

**Figure 1:**
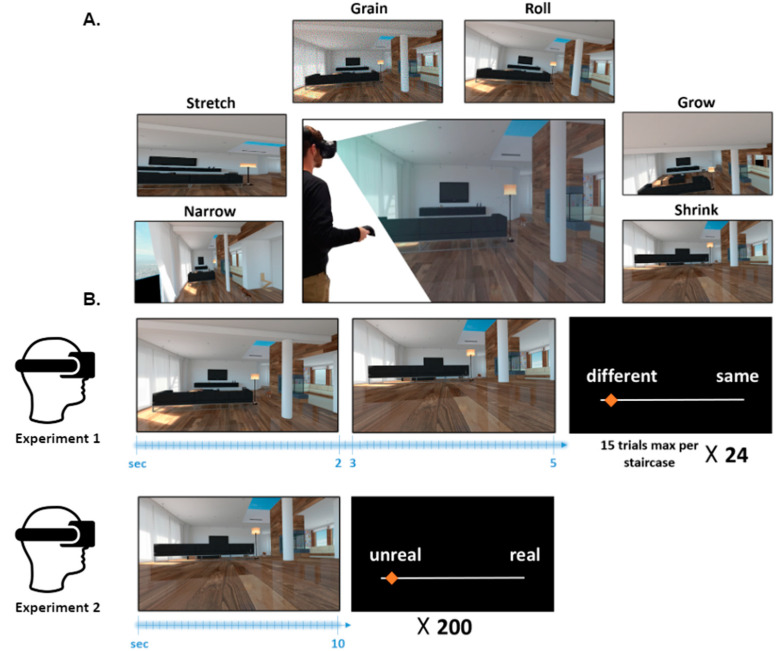
Experimental setup and design. (**A**) Center, illustrative image of the experimental setup and visual scenario. Participants donned a head-mounted display (HMD) and viewed the immersive virtual environment in 360° around them. The images on the sides and bottom represent the six types of alterations of the visual aspects employed, at the highest magnitude of alteration shown, for comparison, on a similar section of the virtual room. (**B**) Trial flow for the experiments. (Top) In Experiment 1, a virtual reality (VR) environment appeared for 2 s, followed by a black screen and then a second VR environment. Subsequently, participants were presented a question screen asking them to report if the two VR environments were identical or not. (Bottom). In Experiment 2, participants viewed a VR environment, which could be altered or unaltered. In each trial, they were required to judge on a continuous scale how ‘real’ the environment felt to them.

SoR was previously challenging to test experimentally as visual manipulations of reality were limited to specific instances, such as prism glasses or still image manipulations (e.g., [34,35]). Here, we developed and tested a novel ecological approach to the study of the SoR, using immersive virtual reality (VR). Virtual reality is now widely employed in scientific studies [36,37] and can be used to manipulate variables that could not otherwise be manipulated (e.g., [38,39]). We created a realistic immersive environment (*UnReal*, Figure 1) in which we can manipulate different visual aspects of reality, creating hallucination like visual stimuli. Importantly, the virtual reality environment allows us to parametrically manipulate such aspects, enabling us to alter reality slightly or massively. For example, we can reduce the height of the participants’ first person viewpoint on the world (*shrink* condition) or increase it (*grow* condition), inducing experiences of changes of *self*, which occur in hallucinatory states (e.g., *Alice in Wonderland* syndrome [40]). Furthermore, we can also induce minute changes, which are barely noticeable by the participants, thus resembling derealization-like states.

We selected several aspects of reality to be examined in this project, based on the phenomenology of distorted visual reality as found in psychiatric, neurological, medical, and pharmacological states [11,12,41]. These alterations of reality broadly fall into three domains: (1) *Perceptual* changes, in which the visual appearance (e.g., graininess) of the scene is manipulated; (2) laws of *nature*, in which we manipulate the visual aspects of the laws of nature (e.g., stretching of the physical world); and (3) changes of *self*, in which we manipulate the participants’ sense of self through conflicts between visual signals and self-related information (e.g., changes in the first person perspective). Alterations of reality in these domains are hallmarks of the phenomenology of hallucinations in psychedelic, neurological, and psychiatric states [42,43,44].

### 1.5. The Present Study: Goals and Predictions

We report the results of two experiments investigating SoR and psychotic symptoms using immersive VR. In experiment 1, we tested the objective psychophysical sensitivity of participants to such alterations by measuring the just noticeable differences (JNDs) between altered and unaltered environments and how these are related to self-reported psychosis symptoms. In experiment 2, we tested the impact of these alterations on the subjective experience of participants, by having them make explicit reality judgments. We hypothesized that psychophysical measures of sensory processing (JNDs) would be stable within participants (low within-subject variance), demonstrating that SoR exhibits robust psychometric properties. Furthermore, we examined whether these measures of SoR are correlated to self-reported prodromal psychotic symptoms, especially for manipulations of the self that are known to be linked to schizophrenia spectrum psychosis [25,27,45]. Finally, we hypothesized that alterations of reality would consistently reduce subjective reality judgments, and this decrease will be related to the magnitude of alteration. Thus, by creating hallucination-like sensory experiences in virtual reality, we examined the impact of parametric induction of distortions on objective and subjective measures of SoR. 

## 2. Methods

### 2.1. Participants

Thirty healthy participants took part in two experiments: Fifteen participated in experiment 1 (10 women, mean age 24.8 years, *SD* = 4.3 years) and 15 in experiment 2 (6 women, mean age 26.6 years, *SD* = 3.8 years). All of them were naïve to the purpose of the experiment, had normal or corrected-to-normal vision, and no self-reported psychiatric or neurological history. All participants gave written informed consent and received payment for their participation (40–50 NIS/~$15 US). The study was performed in accordance with the ethical standards of the Declaration of Helsinki, and the ethics committee of the Gonda Multidisciplinary Brain Research Center approved the experimental protocols.

### 2.2. Hardware

Both experiments were performed on an Intel core i7 processor and 32 GB of RAM computer running in-house software (*UnReal*, built using Unity 2018.3.2). The participants wore a head-mounted display (HMD-HTC Vive) during the experiment. Motion tracking was performed using the HTC VIVE (1.0) system. Subjects responded using the HTC VIVE touch sensitive controller (all VR hardware was manufactured by Valve Corp., Washington, DC, USA). 

### 2.3. Experimental Design

To test the SoR experimentally, we constructed an immersive virtual environment in which we could experimentally manipulate different aspects of reality in a highly controlled fashion (*UnReal*). Here, we used an indoor variant of the *UnReal* environment modeled as an apartment with a high polygon and realistic appearance. The environment contained numerous objects and furniture as well as an animated cat (see Figure 1A for example). The participants were positioned at the center of the room, and observed the environment in 360° from a stationary point. We used a within-subject design in which all participants in each experiment underwent the same experimental protocol.

### 2.4. Virtual Environment

Unity 2018.3.2 was used to construct an environment that would reflect a normative space. The use of a Polybox’s Lounge & Kitchen Pack Asset from Unity Asset Store Environment with real depth and width processing was selected, and additional furniture and accessories were added to make the room realistic. The room’s width, length, and height were 13.2, 24.7, and 3.36, respectively, in the Unity unit system. The camera position was ~8.5 from the right wall when the camera was facing the TV, and 11.5 units from the TV itself.

### 2.5. Alterations of Reality

In order to test the impact of altered reality, we took advantage of the possibilities of VR to induce specific and well-controlled alterations of different aspects of visual reality. Specifically, we manipulated three domains of reality using six aspects (Figure 1A). (1) In the perceptual domain, we manipulated: (a) Graininess of the visual display (*grain*), and (b) the degree of the tilt of the virtual space (*roll*); (2) in the laws of nature domain: (c) Stretching and (d) narrowing of the width dimension of the virtual space (*stretch* and *narrow*); and (3) in the self domain: (e) Elevating and (f) lowering the participants’ first person perspective (*grow* and *shrink*). As mentioned above, these specific alterations were selected based on the phenomenology of distorted visual reality as found in psychiatric, neurological, medical, and pharmacological states and their applicability within immersive virtual reality (see Supplementary Material and Figure S1 for technical details of the implementation of the alterations).

### 2.6. Experiment 1: Psychophysics of Virtually Altered Reality

#### 2.6.1. Experimental Procedure

To assess SoR, in each trial, participants were successively immersed in two virtual environments (2-s duration each), with a black screen displayed (1-s duration) between them (Figure 1B). Critically, the two environments were identical except that one of the environments included an alteration in one of the aspects of reality mentioned above, whereas the other environment was unaltered. Participants then judged whether the environments were ‘different’ or ‘same’ in a classic psychophysics two-alternative forced choice (2AFC) paradigm. We employed a 1-up/ 2-down staircase procedure [46] to derive the JND for each of the aspects of reality. After each response, the alteration level either increased (if the participant judged environments as identical) or decreased (if the participant judged environments as different), by 40% of the current alteration. There were 6 such staircase procedures (one for each condition), which appeared four times each, thus totaling in 24 randomly intermingled staircases each containing only one type of alteration.

Preceding the experimental task, participants were instructed regarding the use of the VR system, the response controllers, as well as the course of the experiment. Then, they performed a training session in which they were acclimated to the VR environment, the experimental task, and response controllers, which lasted until they reported acclimation to the task (26 trials max). To avoid VR motion sickness effects [47], they were instructed to report any discomfort and could stop the experiment at any stage.

#### 2.6.2. Questionnaires

Following task completion, participants completed two self-report questionnaires. The *Cardiff Anomalous Perceptions Scale* (CAPS) and the *Prodromal Questionnaire Brief Version* (PQ-B). The CAPS is a 32-item validated and reliable questionnaire of perceptual anomalies, with subscales of distress, intrusiveness, and frequency of anomalous perceptual experiences [48,49]. The PQ-B primarily serves as an initial screener questionnaire for prodromal or early psychotic symptoms. The PQ-B examines the existence of thoughts and experiences that describe cardinal symptoms of psychosis, such as suspicion, grandiosity, disorganized communication, unconventional thinking, disruptions in perception, difficulties in social functioning, and difficulties in academic or occupational functioning [50,51].

### 2.7. Experiment 2: Subjective Assessment of Virtually Altered Reality

#### Experimental Procedure

Experiment 2 examined the effects of altered reality on participants’ subjective phenomenological experience of reality. In each trial, participants were placed in the center of the virtual environment and instructed to explore it in 360° degrees while standing in one spot. To ensure exploration, they were instructed to search for a target object (a teddy bear). Each trial lasted 10 s or until participants found the object, after which the room disappeared, and the response screen appeared. Participants provided a subjective rating of how ‘real’ or ‘unreal’ the environment felt on a continuous scale of 0–100 (Figure 1B). Each participant performed 200 trials. Sixty trials displayed the environment without alteration, while 140 trials included an alteration of reality in one of the aspects manipulated in experiment 1. Importantly, in each trial, only a single aspect of reality was manipulated. For each aspect of reality, there were four magnitudes of alteration (Supplementary Material Figure S1), which were identical across participants and whose values were based on a pilot study. Each magnitude for each aspect occurred five times, and the order was pseudo-randomized.

Similar to experiment 1, participants were first instructed regarding the use of the VR system, the response controller, and exposed to the environment and equipment until they felt comfortable. Then, they performed a training block of 15 trials, which were excluded from analysis, which included 3 trials with a large change in reality, 2 with mid-level magnitudes of alterations, and 10 with no alterations to practice responses. 

### 2.8. Data Analysis

#### 2.8.1. Experiment 1

Data was processed using in-house Matlab scripts [52]. Statistical analyses were done using JASP 0.11 [53]. To calculate JNDs, for each participant, we averaged the last five trials of each staircase to compute the mean parameter values that the staircase converged on. For each aspect of alteration, these JND values were used to calculate the variance and mean of the JNDs per condition, across the staircase procedures. Conditions with negative numerical scales (e.g., *shrink, narrow*) were converted to absolute values so that all JNDs represented the absolute distance from zero (i.e., from the unaltered condition). It should be noted that comparison across aspect ratings in both experiments was meaningful only for pairs of aspects that shared a similar scale. Therefore, *grow* and *shrink* JNDs were compared using a paired t-test and null effects were assessed using the Bayesian paired t-test, while the remaining aspects could not be directly compared. The within-subject variance of the JNDs in each aspect was calculated across the four staircases. The rate of convergence for each aspect was calculated by averaging the number of steps it took in each staircase until convergence. Pearson’s *r* was used to test for correlations between JNDs across the different aspects. Spearman’s Rho was used to test for correlations between JNDs and the CAPS and PQ-B questionnaires. 

#### 2.8.2. Experiment 2

The subjective ratings of reality in experiment 2 were averaged across participants for each magnitude of manipulation and each aspect separately. These mean values were then used for observing the change in reality ratings across ascending levels of reality manipulations in a repeated measures ANOVA (where normality was violated, a non-parametric Friedman test was performed and Greenhouse–Geisser corrections were applied when required). Pearson’s *r* was used to test for correlations between subjective ratings of reality across aspects. Where possible, we compared subjective judgments using a paired t-test on the difference between the *real* and first level of alteration (which corresponded to the largest reduction in subjective judgements).

## 3. Results

### 3.1. Experiment 1

The average JNDs and their within-subject variance can be seen in Table 1 (see Table S3 for full descriptive statistics). 

**Figure 2:**
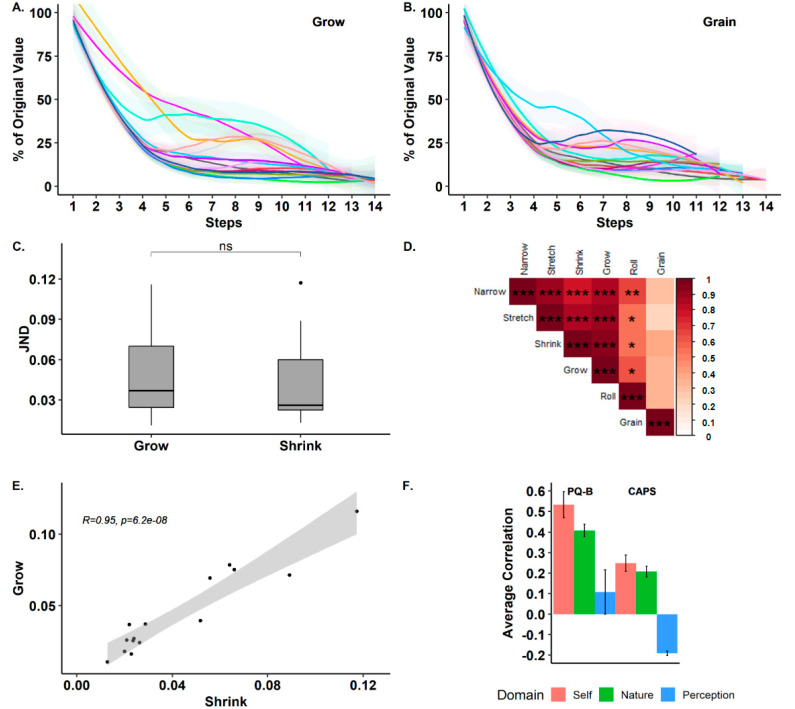
Perceptual thresholds for reality alterations. (**A**,**B**). Convergence rates of the staircase procedure in aspects *grow* and *grain*, respectively. *Y*-axis shows the magnitude of the reality alteration and the *x*-axis denotes the number of iterations. Note individual participants (colored lines) converged towards a stable perceptual threshold. (**C**) A comparison between JNDs of *grow* and *shrink* aspects that shared a common scale. (**D**) Pearson correlation matrix between JNDs across all aspects. Note high, positive, and significant correlations were found between all aspects’ JNDs, with the exception of *grain*. (**E**) Example of correlation between participants’ *grow* and *shrink* JNDs. (**F**). Averaged correlations of The *Cardiff Anomalous Perceptions Scale* (CAPS) and the *Prodromal Questionnaire Brief Version* (PQ-B) scores with JNDs shown by aspect domains (i.e., self, nature, perception).

Overall, participants showed very stable JNDs with little within-subject variability. For example, the average JND for *narrow* was (*M* = 0.09) and the average within-subject variability was (*SD*^2^ = 0.003). In *stretch*, the average JND was (*M* = 0.12) and the average within-subject variance was (*SD*^2^ = 0.004). The exception to this low variance was *roll*, which showed larger variance: (*M* = 2.57, *SD*^2^ = 0.93). Thus, the average variability of the JNDs for all but one condition was approximately ~2.5% of the mean JND (8.2% including *roll*). This demonstrates the visual sensitivity to changes across different aspects of reality alteration and their relative consistency within participants. The *roll* condition showed greater within-subject (and between-subject) variability. We suspect this may have occurred due to some participants’ compensating for the visual manipulation of *roll* by tilting their heads in the opposite direction. In addition to the consistency of the JNDs themselves, the number of trial steps needed for the staircase procedure to converge was also similar across aspects. Convergence rates ranged from 5.7 to 8.7 (trial steps) across conditions. For instance, the average *grow* and *grain* convergence rates were (*M* = 6.5, *SD* = 1.98; *M* = 6.7, *SD* = 1.76), respectively. The average convergence rate across all aspects was (*M* = 6.8, *SD* = 1.05), indicating that our staircase procedure was effective at uncovering perceptual thresholds across conditions (see Figure 2A,B. for examples of convergence rates for *grow* and *grain* and Table 1 for all conditions). Combined, the measure of within-subject variability along with the consistent convergence rate of the staircase procedures implies that perceptual thresholds of SoR exhibit stable psychometric properties that can be measured experimentally.

Examining the correlations between JNDs across the different aspects (see the correlation matrix in Figure 2D and the example correlation in Figure 2E), they exhibited high, positive, and significant correlations ranging from 0.22 to 0.95 (*M* = 0.61, *SD* = 0.25). Thus, participants with high sensitivity in one aspect were also likely to be sensitive in another aspect and vice versa. An outlier to this pattern was the *grain* aspect, which showed low levels of correlations with all other aspects (*M* = 0.31, *SD* = 0.05). Next, directly comparing JNDs for the *grow* and *shrink* aspects, which share a common parameter scale (see Figure 2C), a paired t-test revealed no significant difference between the two (*M_Grow_* = 0.05, *SD_Grow_* = 0.03; *M_Shrink_* = 0.04, *SD_Shrink_* = 0.03, *t*(14) = −0.66, *p* = 0.51, *Cohen’s d* = −0.17). Bayesian analysis provided moderate evidence (*BF*_10_ = 0.31) for there being no difference in the perceptual sensitivity for vertical changes in both directions.

Clinical questionnaires (CAPS and PQ-B) showed low overall scores (*M_CAPS_* = 3, *SD_CAPS_* = 4.24, *M_PQ-B_* = 2.8, *SD_PQ-B_* = 3.78) as expected in a non-clinical cohort (see Table S2 for detailed CAPS and PQ-B scores with subscales) [48,50]. Importantly, however, an analysis of the correlations between CAPS and PQ-B, with perceptual sensitivity to alterations, found that JNDs in the *grow* condition were significantly correlated with the PQ-B general score (*Spearman’s Rho* = 0.61, *p* = 0.015), indicating that participants showing higher levels of psychotic symptoms also had reduced discrimination between ‘real‘ and ‘unreal‘ perceptions of *self.* Furthermore, the *grow* condition showed a high positive but non-significant (*Spearman’s Rho* = 0.45, *p* = 0.09) correlation with the CAPS general score, indicating that reduced sensitivity to changes in the first person perspective (1PP) is related to abnormal perceptual experiences (see Table S1 for the full correlation matrix with subscales). 

### 3.2. Experiment 2 (Subjective Reality Rating)

**Figure 3:**
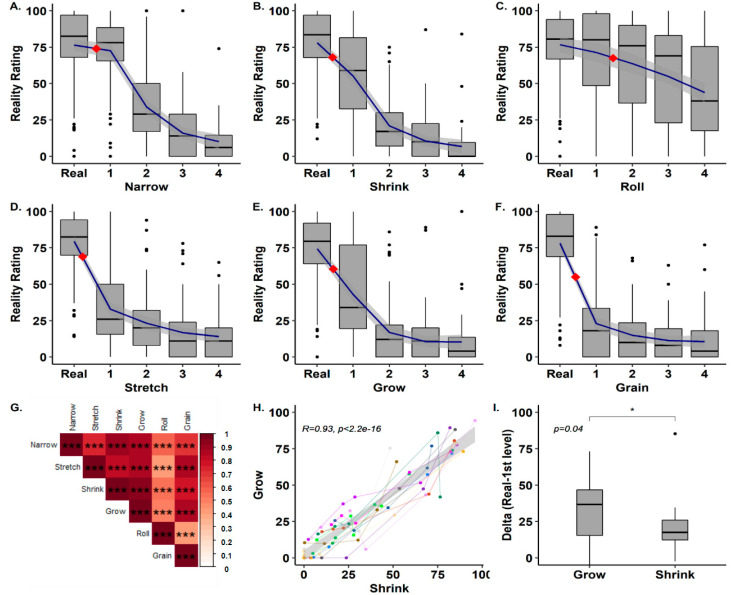
Subjective judgments of reality. (**A**–**F**) Changes in subjective reality affected the ratings of Sense of Reality (SoR) in a relatively consistent manner. Reality ratings for unaltered environments were highest while ratings for altered environments were reduced, suggesting a consistent tuning curve for SoR. (**G**) Subjective ratings for all aspects showed high, positive, and significant correlations. (**H**) Example of the correlation between participants’ subjective rating of *grow* and *shrink*. (**I**) Significant difference between the drop in subjective ratings for *grow* and *shrink*, indicating an asymmetrical response for inducing changes in the first person perspective (1PP).

The analysis of subjective reality ratings revealed several findings. First, for the unaltered condition (*real*), the mean reality ratings were the highest (*M* = 77.21, *SD* = 12.32), demonstrating the validity of our experimental paradigm. Second, increasing magnitudes of alteration reduced the subjective reality ratings significantly for all aspects. For example, in the *grow* aspect, ratings dropped from (*M* = 75, *SD* = 13.99) in the unaltered condition to (*M* = 9.57, *SD* = 8.47) in the largest alteration magnitude, with a repeated measures ANOVA revealing a significant difference in ratings across alteration magnitudes (*F_Grow_*(2.39,14) = 79.32, *p_Grow_* < 0.001, *η*^2^*_Grow_* = 0.77). All other aspects showed similar patterns: (*χ^2^_Shrink_*(4) = 54.15, *p_Shrink_* < 0.001, *η*^2^*_Shrink_* = 0.8; *χ^2^_Grain_*(4) = 40.05, *p_Grain_* < 0.001, *η*^2^*_Grain_* = 0.8; *χ^2^_Roll_*(4) = 20.32, *p_Roll_* < 0.001, *η*^2^*_Roll_* = 0.22; *F_Stretch_*(4,14) = 91.91, *p_Stretch_* < 0.001, *η*^2^*_Stretch_* = 0.78; *F_Narrow_* (2.63,14) = 136.15, *p_Narrow_* < 0.001 *η*^2^*_Narrow_* = 0.84). Figure 3A–F show the average change in the ratings of subjective reality in all aspects for all levels of alteration. The *roll* condition showed a more linear reduction of reality judgments across the selected magnitudes. We note, however, that in the current design, the manipulations in different aspects are on different scales (based on a pilot experiment) and thus comparisons between different aspects are not possible, apart from the *grow* and *shrink* conditions. We thus compared the mean reduction in subjective ratings in *grow* and *shrink* between the *real* and first alteration magnitude (as this included the largest decrease in ratings across all conditions). Interestingly, in contrast with the similarity of their JNDs, a paired t-test indicated that the initial drop in reality ratings was significantly larger for the *grow* condition (*M_Grow_* = 32.12, *SD_Grow_* = 23.19) than for the *shrink* condition, (*M_Shrink_* = 21.18, *SD_Shrink_* = 20.81, *t*(14) = −2.26, *p* = 0.04, *Cohen’s d* = −0.58, Figure 3I). Indicating that while objective perceptual sensitivity was similar, identical modulations of 1PP in the *grow* and *shrink* conditions had a differential impact on subjective reality judgments (see Table 2 and Table S4 for full descriptive statistics of the subjective ratings of reality across aspects and levels).

Third, the correlations in the reality ratings between different aspects of alteration were high, positive, and significant. Correlations between aspects ranged from 0.42 to 0.93 (*M* = 0.72, *SD* = 0.17, Figure 3G), indicating that participants’ subjective judgments were similar across the different aspects. That is, participants judging the environment to be unrealistic in one aspect were also likely to do so in another aspect. 

Finally, despite the fact that experiments 1 and 2 were conducted in different cohorts, we were curious regarding the relation between the objective sensitivity to alterations of reality (JNDs) and the effects of these alterations on subjective judgments. To this end, we superimposed the average JND for each aspect onto their respective average subjective ratings plot. Interestingly, for most aspects, the average JNDs (i.e., the perceptual sensitivity to a change in this aspect of reality) was found between *real* and the first magnitude of reality alteration (red diamonds in Figure 3A−F). The *roll* condition was an exception to this, with the JND found between the first and second alteration magnitudes (Figure 3C). Thus, in several aspects, the mean perceptual threshold corresponded to the point in which subjective judgments were massively reduced (e.g., *stretch*, Figure 3D). However, other aspects showed a more graded reduction of subjective reality judgments taking place at magnitudes larger than the liminal perceptual level (e.g., *narrow*, Figure 3A).

## 4. Discussion

SoR is a fundamental and ubiquitous criterion in assessing mental health, but it has been difficult to assess experimentally. This is mainly due to the lack of experimental paradigms allowing for well-controlled manipulations of reality. Employing immersive VR and a novel methodological framework, we demonstrated that both objective and subjective measures of SoR can be studied experimentally and that these show potentially meaningful correlations to clinical measures of psychosis. 

### 4.1. The Psychophysics of SoR

In the first experiment, we examined the objective perceptual sensitivity (JNDs) to different aspects of altered reality. Our results revealed several interesting findings. First, objective perceptual thresholds for detecting alterations of reality (JND) were highly consistent within subjects, with within-subject variability in most aspects on an order of (~2.5%) of the mean JND magnitude. In addition, convergence rates were also similar across aspects. These findings suggest that JNDs for the alterations of reality are a robust and replicable psychometric measure. Furthermore, the high and significant correlations between all aspects, apart from *grain*, suggest that the perceptual thresholds across the conditions may rely on similar cognitive processes. Contrarily, JNDs in the *grain* condition were not significantly correlated with JNDs in the other conditions, suggesting that it may have been achieved in a different manner. Importantly, the PQ-B questionnaire responses, probing prodromal symptoms, were highly correlated with perceptual thresholds in the *self* domain (Figure 2F). This indicates that participants with higher ratings of psychosis-like experiences also showed lower sensitivity to changes in manipulation of the first person perspective. This finding is especially interesting given the well-established link between altered self-related processing and psychosis [25,27,45,54]. Similarly, JNDs in the *self* domain were also positively, but non-significantly, correlated with the CAPS questionnaire scores, providing converging evidence for the relationship between perceptual thresholds for reality alterations and clinical symptoms. These correlations, found in healthy participants with low levels of symptoms, are likely to be accentuated in clinical populations, and provide preliminary evidence for the viability of SoR as a diagnostic criterion.

### 4.2. Subjective Modulation of SoR

The second experiment measured the impact of alterations of reality on subjective judgments of reality. As predicted, we found that when the virtual environment was unaltered, most participants reported higher levels of reality judgments (*M* = 77.21, *SD* = 12.32). Furthermore, increasing magnitudes of reality alterations reduced the subjective judgments of reality (Figure 3A–F). However, as can be seen in Figure 3A–F, there were several outlier participants, which gave extremely low ratings during the *real* condition. It seems that these participants did not accept the basic premise of the task (e.g., accepting the baseline unaltered VR environment as realistic). We note that our results are robust and significant despite this; however, future experiments may benefit from prescreening for such participants.

The different aspects of reality modulated showed different rates of decrease as a function of the alteration magnitude. We note, however, that the current experiment does not allow direct comparisons between the different aspects as they use different scales (e.g., angle for the *roll* condition and percentage of height for *grow*). However, *grow* and *shrink* used the same measure and scaling factor, allowing a direct comparison between them. As predicted, when the participants’ 1PP was elevated (*grow*) this reduced reality judgments significantly more than a similar magnitude of 1PP reduction (*shrink*, Figure 3I). This finding is compatible with a predictive coding framework, and specifically the notion that alterations for which we have more experience will have a smaller impact on SoR. Given that we have more experience with the reduction of our 1PP (through sitting and laying down); this had less impact on SoR compared with the *grow* condition for which we have less experience. Indeed, 1PP is considered a fundamental component of the sense of self [43]. Changes in the bodily-self have been found in both neurological (e.g., [55]) and psychiatric [27,56] conditions in which hallucinations are prevalent. Furthermore, experimentally induced changes in the sense of self impact perceptual awareness and self-consciousness [57,58,59]. 

Comparing the mean JNDs from the first experiment with the subjective judgments from experiment 2 revealed that for some aspects, the JND marked the point of the greatest reduction in subjective reality (i.e., *stretch*, *grow*, and *grain*). Thus, the perceptual threshold coincided with the subjective experience of reality, as one may expect. However, in other aspects, the largest reduction occurred at larger alteration magnitudes than the JND (i.e., *shrink, roll*, and *narrow*). This suggests that subjective judgments are not completely dependent on the objective threshold in certain aspects of reality. We therefore propose that JNDs can be used as standardized units to allow comparisons of SoR across the different aspects. Future experiments will capitalize upon this finding by employing participants’ JNDs to normalize the levels of alterations across conditions. In turn, this will allow comparison of the impact of different alterations of reality, allowing the modeling of one’s implicit model of expectations of the world. For example, this method would allow us to compute how a change in the self (e.g., 1PP modulation—*grow*) compares to an alteration in a more perceptual aspect (e.g., *grain*), as they will share a common scale.

The broader implications of this study may potentially go beyond providing a novel approach for investigating mechanisms of SoR. The current paradigm (*UnReal*) may allow both laboratory and online gathering of large data sets in diverse cohorts, enabling a starting point for a mechanistic model of SoR. The availability of a reliable mechanistic model of SoR and its clinical and behavioral correlates is bound to provide researchers and clinicians with a new set of conceptual as well as practical tools to address research questions in a large variety of neuropsychiatric and neurological disorders involving hallucinatory or dissociative symptoms. In turn, this may lead to useful measures to further support clinical diagnoses and enable clinicians to develop and employ more accurately targeted treatments, such as new neurocognitive markers for the early detection of psychosis. Finally, our paradigm may allow for the development of novel VR-based therapeutic interventions that enhance and restore SoR in clinical populations with deficits of SoR, similar to approaches in other neurological and psychiatric conditions [60,61,62].

### 4.3. Limitations

The current experiments were aimed to test our novel approach to the study of SoR and its relation to psychosis. A central limitation here is that the objective and subjective measurements of SoR were conducted in separate cohorts, thus direct within-subject comparisons were not possible. Future studies will employ both tasks within the same cohort. Furthermore, the PQ-B and CAPS questionnaires were used only in the first experiment, thus the relationship between subjective judgments and clinical symptoms of psychosis and hallucinations is yet to be assessed. Finally, both studies had low numbers of participants and thus suffered from low statistical power, which in turn limited our ability to examine individual differences, such as the effects of age and gender. Further, higher powered studies including clinical populations are needed to validate and extend these findings regarding SoR and psychosis.

### 4.4. Summary

The present study investigated the SoR and its relation to psychotic symptoms. By inducing hallucination-like visual experiences and testing objective and subjective measures of SoR, we found a novel psychophysical link between sensitivity to alterations of reality and prodromal psychotic symptoms. These results provide evidence for the utility of this ecological and immersive VR methodology for the scientific study of SoR and a novel tool for psychiatric clinical assessment. Future studies may employ the *UnReal* paradigm to build computational models as well as investigate the neural substrates of SoR in healthy and clinical populations. As SoR is a central benchmark in determining psychiatric and neurological wellbeing, it is critical that we acquire a fuller understanding of how SoR is constructed by the brain and mind, allowing us to provide better diagnostic and therapeutic tools. 

## Figures and Tables

**Table 1:** Descriptive statistics for experiment 1.

	**JND**					
	**Narrow**	**Stretch**	**Shrink**	**Grow**	**Roll**	**Grain**
**Mean**	0.09	0.12	0.04	0.05	2.57	0.01
**Std. Deviation**	0.06	0.06	0.03	0.03	2.37	0.05
**Minimum**	0.04	0.07	0.01	0.01	0.31	0.03
**Maximum**	0.24	0.29	0.12	0.12	9.5	0.19
	**Within-Subject Variability**					
	**Narrow**	**Stretch**	**Shrink**	**Grow**	**Roll**	**Grain**
**Mean**	0.003	0.004	0.001	8.0e -4	0.93	0.002
**Std. Deviation**	0.003	0.009	0.002	0.002	1.05	0.003
**Minimum**	0	0	0	0	0.003	0
**Maximum**	0.008	0.04	0.007	0.006	3.92	0.01
	**Convergence Rate**					
	**Narrow**	**Stretch**	**Shrink**	**Grow**	**Roll**	**Grain**
**Mean**	5.99	8.73	7.03	6.57	5.74	6.76
**Std. Deviation**	1.47	0.91	1.6	1.98	2.23	1.76
**Minimum**	3	7	4	3	1	4
**Maximum**	9	10	9.5	9.75	10	10

**Table 2:** Descriptive statistics for experiment 2.

Subjective Ratings												
Alteration Magnitude	Narrow		Stretch		Shrink		Grow		Roll		Grain	
	Mean	SD	Mean	SD	Mean	SD	Mean	SD	Mean	SD	Mean	SD
**Real**	76.84	10.42	79.42	14.34	76.26	15.85	75	13.99	76.96	12.95	78.78	12.51
**1**	72.59	12.68	32.88	14.46	55.08	19.4	42.88	18.35	71.41	17.7	22.99	16.72
**2**	33.89	16.27	23.2	13.8	20.96	10.98	16.93	13.2	63.68	25.19	14.95	13.24
**3**	17.92	11.54	16.67	12.5	14.31	11.67	13.68	12.4	55.2	28.97	11.65	10.61
**4**	9.63	8.59	14.09	9.015	5.76	7.92	9.57	8.47	43.79	24.82	10.47	12.02

## Supplementary Materials

The following are available online at https://www.mdpi.com/2077-0383/9/6/1627/s1, Figure S1: Visual display of alterations, Table S1: Correlation matrix of JNDs and clinical questionnaires, Table S2: Descriptive statistics of Questionnaire Responses. Table S3. Descriptive statistics of JNDs across aspects, Table S4. Descriptive statistics of Subjective ratings across aspects.
