# Supplementary material for: UnReal? Investigating the Sense of Reality and Psychotic Symptoms with Virtual Reality
Source: J Clin Med. 2020 May 28;9(6):1627. doi: 10.3390/jcm9061627 (PMC7355917; doi:10.3390/jcm9061627)
Supplement: Supplementary file 1 [file jcm-09-01627-s001.pdf]

Supplementary Materials

Methods

Creation of Manipulated Visual Parameters

Shrink & Grow

Two game objects were placed - one that marked the floor and one that marked the ceiling. With regard to Steam VR Player, the difference between the two objects was calculated using a script written in Microsoft Visual Studio. This difference was multiplied by a parameter (T), representing a percentage of the difference, that was changed according to the intensity of manipulation required. According to this equation, the height of the camera in the environment could be adjusted regardless of the participant's height. For example – for level 1 in *Grow*, T was selected to be 0.6, locating the camera at 60% of the room height, with the Real level being 50% - in the middle of the room height.

Grain

Post Process Stack plugin, version 2.1.6 of type "Grain" was used. The following properties: size, intensity and luminance, could be arbitrarily altered. For the experiments, only the grain intensity attribute was used while the size and the luminance contribution remained unchanged (3 and 0, respectively). At the Real level the value of the intensity was 0.

Roll

The room rotates with its pivot placed on the game object that marked the floor. We used the following function:

gameObject.transform.Rotate (-1 * T * Vector3.right, Space.self)

When -1 determines the slope direction, and T determines the tilt in degrees. The default T value (at the Real level) was 0.

Stretch & Narrow

These alterations were made with reference only to *distort X*. Using the same pivot on the Room placed on the floor, we used the function:

gameObject.transform.localScale(T,1,1).
In the default state (real level) T = 1.

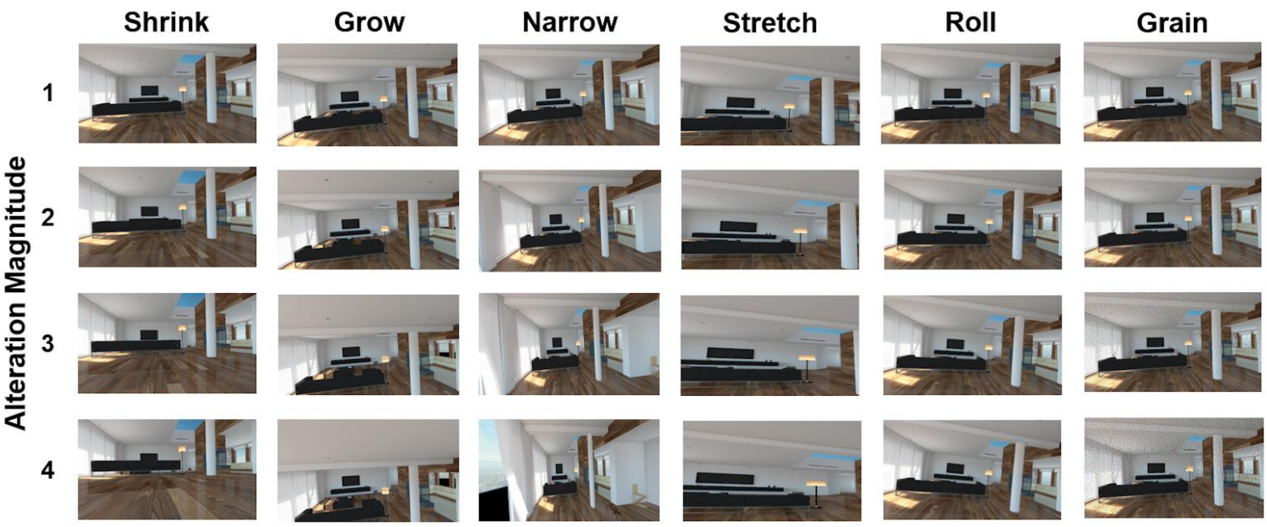

Figure S1. Visual display of alterations.

Each parameter was divided into four levels with the 1st level representing the minimal alteration of an aspect and the 4th level representing the maximal alteration. The different levels for four out of the six aspects are shown by row. Note, that the presented aspects do not share the same scales; therefore the difference between two levels cannot be compared between aspects (with the exception of *grow* & *shrink*). Additionally, it should be noted that the division into levels in each aspect is arbitrary and based on results from a previous pilot study.

Table 1. Correlation matrix of JNDs and clinical questionnaires.

PQB, CAPS and JND Correlations

Spearman Correlations

		Narrow	Stretch	Shrink	Grow	Roll	Grain	PQB_General	PQB_Distress	CAPS_General	CAPS_Distress	CAPS_Intrusivness	CAPS_Frequency
Narrow	Spearman's rho	—											
	p-value	—											
Stretch	Spearman's rho	0.611*	—										
	p-value	0.018	—										
Shrink	Spearman's rho	0.657**	0.821***	—									
	p-value	0.010	< .001	—									
Grow	Spearman's rho	0.818***	0.700**	0.893***	—								
	p-value	< .001	0.005	< .001	—								
Roll	Spearman's rho	0.689**	0.561*	0.600*	0.657**	—							
	p-value	0.006	0.032	0.020	0.010	—							
Grain	Spearman's rho	0.582*	0.521*	0.550*	0.557*	0.439	—						
	p-value	0.025	0.049	0.036	0.034	0.103	—						
PQB_General	Spearman's rho	0.461	0.365	0.510	0.615*	0.217	0.069	—					
	p-value	0.083	0.181	0.052	0.015	0.438	0.808	—					
PQB_Distress	Spearman's rho	0.412	0.347	0.515*	0.607*	0.186	0.085	0.992***	—				
	p-value	0.127	0.205	0.049	0.016	0.506	0.763	< .001	—				
CAPS_General	Spearman's rho	0.179	-0.032	0.329	0.451	-0.080	0.046	0.548*	0.605*	—			
	p-value	0.524	0.909	0.231	0.092	0.777	0.872	0.035	0.017	—			
CAPS_Distress	Spearman's rho	0.252	0.026	0.376	0.497	-0.075	0.087	0.565*	0.613*	0.986***	—		
	p-value	0.364	0.926	0.167	0.059	0.790	0.759	0.028	0.015	< .001	—		
CAPS_Intrusivness	Spearman's rho	0.260	0.017	0.371	0.490	-0.087	0.096	0.556*	0.603*	0.982***	0.999***	—	
	p-value	0.349	0.952	0.173	0.064	0.759	0.733	0.031	0.017	< .001	< .001	—	
CAPS_Frequency	Spearman's rho	0.258	0.006	0.367	0.488	-0.030	0.051	0.590*	0.639*	0.987***	0.987***	0.986***	—
	p-value	0.353	0.984	0.178	0.065	0.915	0.857	0.021	0.010	< .001	< .001	< .001	—

* p < .05, ** p < .01, *** p < .001

Table S2. Descriptive statistics of Questionnaire Responses.

	PQB General	PQB Distress	CAPS General	CAPS Distress	CAPS Intrusiveness	CAPS Frequency
**Mean**	2.800	14.867	3.000	7.533	6.533	4.467
**Std. Deviation**	3.783	19.686	4.243	11.940	10.508	6.760
**Skewness**	1.603	1.411	1.470	1.784	1.917	1.687
**Kurtosis**	2.531	1.536	1.074	2.298	3.087	1.847
**Minimum**	0.000	0.000	0.000	0.000	0.000	0.000
**Maximum**	13.000	65.000	13.000	38.000	35.000	20.000

Table S3. Descriptive statistics of JNDs across aspects.

	JND					
	**Narrow**	**Stretch**	**Shrink**	**Grow**	**Roll**	**Grain**
**Mean**	0.09	0.12	0.04	0.05	2.57	0.01
**Std. Deviation**	0.06	0.06	0.03	0.03	2.37	0.05
**Skewness**	1.85	1.95	1.31	1.04	2.08	0.50
**Kurtosis**	2.93	3.83	1.12	0.42	4.82	-0.12
**Minimum**	0.04	0.07	0.01	0.01	0.31	0.03
**Maximum**	0.24	0.29	0.12	0.12	9.5	0.19
	**Within-Subject Variability**					
**Mean**	0.003	0.004	0.001	0.0008	0.93	0.002
**Std. Deviation**	0.003	0.009	0.002	0.002	1.05	0.003
**Skewness**	0.93	3.39	2.37	2.73	2.15	2.43
**Kurtosis**	-0.13	12.10	5.69	8.25	4.54	6.60
**Minimum**	0	0	0	0	0.003	0
**Maximum**	0.008	0.04	0.007	0.006	3.92	0.01
	**Convergence Rate**					
**Mean**	5.99	8.73	7.03	6.57	5.74	6.76
**Std. Deviation**	1.47	0.91	1.6	1.98	2.23	1.76
**Skewness**	-0.10	-0.76	-0.50	-0.09	-0.52	0.32
**Kurtosis**	1.00	-0.04	-0.57	-0.50	1.02	-0.65
**Minimum**	3	7	4	3	1	4
**Maximum**	9	10	9.5	9.75	10	10

Table S4. Descriptive statistics of Subjective ratings across aspects.

	Grain					Shrink					Grow					Roll					Stretch					Narrow				
	**Real**	**1**	**2**	**3**	**4**	**Real**	**1**	**2**	**3**	**4**	**Real**	**1**	**2**	**3**	**4**	**Real**	**1**	**2**	**3**	**4**	**Real**	**1**	**2**	**3**	**4**	**Real**	**1**	**2**	**3**	**4**
**Mean**	78.77	22.98	14.94	11.65	10.46	76.26	55.08	20.96	14.30	5.76	75.00	42.88	16.93	13.68	9.57	76.96	71.41	63.68	55.20	43.78	79.42	32.88	23.20	16.66	14.09	76.84	72.58	33.89	17.92	9.62
**Std. Deviation**	12.51	16.72	13.23	10.61	12.02	15.85	19.40	10.98	11.67	7.91	13.99	18.35	13.20	12.40	8.47	12.94	17.70	25.19	28.97	24.82	14.34	14.45	13.80	12.49	9.01	10.42	12.67	16.27	11.54	8.59
**Skewness**	-0.18	-0.09	0.47	0.53	0.80	-1.20	-1.44	0.44	0.21	1.82	-1.51	-0.62	0.02	0.79	0.17	-1.12	0.16	-0.74	-0.57	-0.46	-1.04	-0.18	0.09	0.37	1.18	-0.99	0.39	0.54	0.43	0.63
**Kurtosis**	-0.68	-0.48	-1.05	-1.05	-0.97	0.26	2.19	1.61	-1.26	3.79	3.85	1.33	-1.45	0.14	-1.61	0.76	-1.31	0.18	-0.60	-0.76	2.23	-1.41	-1.00	-0.83	2.82	1.70	-0.86	0.87	-0.34	-0.31
**Minimum**	54.87	0.00	0.00	0.00	0.00	43.50	4.00	0.20	0.00	0.00	35.63	0.00	0.00	0.00	0.00	45.57	44.80	7.00	0.00	0.00	42.25	10.00	0.00	0.00	0.00	50.44	53.40	7.00	0.00	0.00
**Maximum**	98.83	55.20	38.80	29.80	31.60	95.88	76.40	47.20	34.80	28.20	94.25	76.80	37.00	41.80	22.80	91.20	100.00	96.40	92.40	78.20	100.00	56.00	47.00	39.80	38.20	92.57	97.00	71.40	40.20	27.40
